# COVID-19 and technological addiction: The role of loneliness

**DOI:** 10.1192/j.eurpsy.2021.1493

**Published:** 2021-08-13

**Authors:** G. Rogier, S. Beomonte Zobel, P. Velotti

**Affiliations:** Dynamical And Clinical Psychology, Sapienza Università di Roma, Rome, Italy

**Keywords:** gaming, loneliness, social network addiction, COVID-19 outbreak

## Abstract

**Introduction:**

The Covid-19 outbreak has shown to negatively impact on mental health. Several anecdotical and theoretical evidences argued that lockdown measures would have increased subjective feelings of loneliness and addictions’ proneness.

**Objectives:**

In addition, preliminary data underlined a possible increase in the frequency of gaming and social media use. Increased loneliness levels are likely to account for increased gaming and social media addiction during the lockdown.

**Methods:**

We conducted a longitudinal study administering to a sample of 154 Italian adults several self-report questionnaires at the beginning of lockdown (Time 1) and three days before the end of the lockdown (Time 2). We therefore assessed loneliness feelings, frequency of gaming and social media use as well as both gaming and social media addiction. Data were analysed using Structural Equation Modelling.

**Results:**

We observed that loneliness levels longitudinally predicted both gaming and social media addiction also controlling for gaming and social media use at Time 1.

**Conclusions:**

Increased feelings of loneliness, a well-known risk factor for gaming and social media addiction, may be a central variable heightening vulnerability to the onset or the maintenance of technological addiction during forced social isolation. Thus, future prevention interventions may want to target this issue.

